# Immune checkpoint inhibitors and myocarditis in advanced non-small cell lung cancer: a nationwide cohort study

**DOI:** 10.1186/s40959-025-00325-6

**Published:** 2025-03-31

**Authors:** Fu-Xiao Li, Jia-Xin Cai, Ji-Bin Li, Kong-Jia Luo, Shi-Yu Wang, Wei-Hua Meng, Feng Sha, Zhi-Rong Yang, Allan Hackshaw, Jin-Ling Tang

**Affiliations:** 1 Department of Computational Biology and Medical Big Data, Shenzhen University of Advanced Technology, Shenzhen, China; 2 Nottingham Ningbo China Beacons of Excellence Research and Innovation Institute, University of Nottingham Ningbo China, Ningbo, China; 3 Department of Clinical Research, Sun Yat-sen University Cancer Center, the State Key Laboratory of Oncology in South China, Collaborative Innovation Center for Cancer Medicine, Guangzhou, China; 4 State Key Laboratory of Oncology in South China, Sun Yat-Sen University Cancer Center, Guangzhou, China; 5 Shenzhen Institute of Advanced Technology, Chinese Academy of Sciences, Shenzhen, China; 6 Department of Public Health and Primary Care, School of Clinical Medicine, University of Cambridge, Cambridge, UK; 7 CRUK & UCL Trials Centre, University College London, 5 Floor, 90 Tottenham Court Road, London, UK

**Keywords:** Non-small cell lung cancer, Immune checkpoint inhibitor, Myocarditis, Cohort study, Real-world evidence, Cardiotoxicity

## Abstract

**Objective:**

Evidence suggests immune checkpoint inhibitor (ICI) can increase the risk of myocarditis. We investigated it in a large national cohort in China.

**Methods:**

Patients with stage IIIB-IV non-small cell lung cancer (NSCLC) using data from China's National Anti-Tumor Drug Surveillance System between January 2013 and December 2021. Exposure density sampling was applied to control for immortal time bias. Multivariate Cox regression with time-dependent exposures was used to examine the association between ICI therapy and the incidence of myocarditis while controlling for confounders.

**Results:**

55,219 patients were included. The median age was 61 years, and 62% were males. At one-year follow-up (median 335 days), there were 26 cases of myocarditis among ICI users and 28 cases among ICI non-users (a cumulative incidence of 4.8 and 0.6 per 1000 person-years respectively). The adjusted hazard ratio (HR) of myocarditis for ICI users was 7.41 (95% confidence interval [CI]: 3.29–16.67). For programmed cell death protein 1 inhibitor users the HR was 8.39 (95% CI: 3.56–19.77). No significant interactions were observed in subgroup analysis. The results remained unchanged in sensitivity analyses.

**Conclusions:**

This study showed that ICI therapy considerably increased the risk of myocarditis, supporting the need for closer monitoring of patients receiving ICI therapies.

**Supplementary Information:**

The online version contains supplementary material available at 10.1186/s40959-025-00325-6.

## What is already known on this topic

Previous evidence showed that myocarditis is a rare and life-threatening adverse event with notably increased risk among users. Myocarditis seemed to occur soon (mostly within 3 months) after initiation of immunotherapy.

## What this study adds

Our study suggested that immunotherapy is associated with a 7-fold increased risk of myocarditis within one year following initiation, and the risk at 6 months after initiation was higher than that at 3 months.

## How this study might affect research, practice or policy

The risk of myocarditis, although rare, was notably increased by immunotherapy in advanced non-small cell lung cancer patients and a longer duration of surveillance for myocarditis after initiation of immune checkpoint inhibitors should be warranted. Further studies are needed to confirm whether the risk varies by type of ICI, and if there is a dose-response relationship.

## Introduction

In the last decade, immune checkpoint inhibitors (ICIs), targeting cytotoxic T lymphocyte-antigen-4 (CTLA-4), programmed cell death protein 1 (PD1), and its ligand 1 (PD-L1), have greatly improved the survival of lung cancer and malignant melanoma [1, 2], and their use has expanded in other types of cancer [3]. However, there are some concerns regarding the possible cardiovascular adverse effects of ICI [4, 5] in particular myocarditis, a life-threatening condition with a mortality rate of around 50% [6–9]. High quality evidence on this topic is lacking.

After a series of case reports on myocarditis events following ICI treatment [9–11], the VigiBase (the World Health Organisation pharmacovigilance database) and the US Food and Drug Administration (FDA) Adverse Event Reporting System (FAERS) database studies have highlighted this issue [6, 12]. The only observational cohort study among lung cancer patients who were given ICI therapy reported a significant increase in the risk of myocarditis or peri-myocarditis [hazard ratio = 5.51, 95% CI (2.85–10.66)] but there were only 11 cases [13]. However, results from meta-analyses of randomized controlled trials were inconsistent [14, 15] due to the differences in inclusion criteria. One found no association among 24,156 patients [17 cases of myocarditis, hazard ratio = 1.11, 95% CI (0.64–1.92)] [14], but the other reported a significant association among 9,455 patients [15 cases of myocarditis, Peto’s OR = 4.42, 95% CI (1.56–12.50)] [15]. Racial / ethnic disparities in ICI-related adverse events have also been observed in trials but Asian populations including Chinese were underrepresented [16, 17]. In addition, there were various methodological flaws in these prior studies, notably in the selection of participants, definition of outcomes, and control for immortal time bias and confounding factors.

We thus analysed a large nationwide cohort study with long follow-up using the main database in China to examine the association between ICI use and incident myocarditis among advanced non-small cell lung cancer (NSCLC) patients.

## Methods

### Data source and study population

**Fig. 1:**
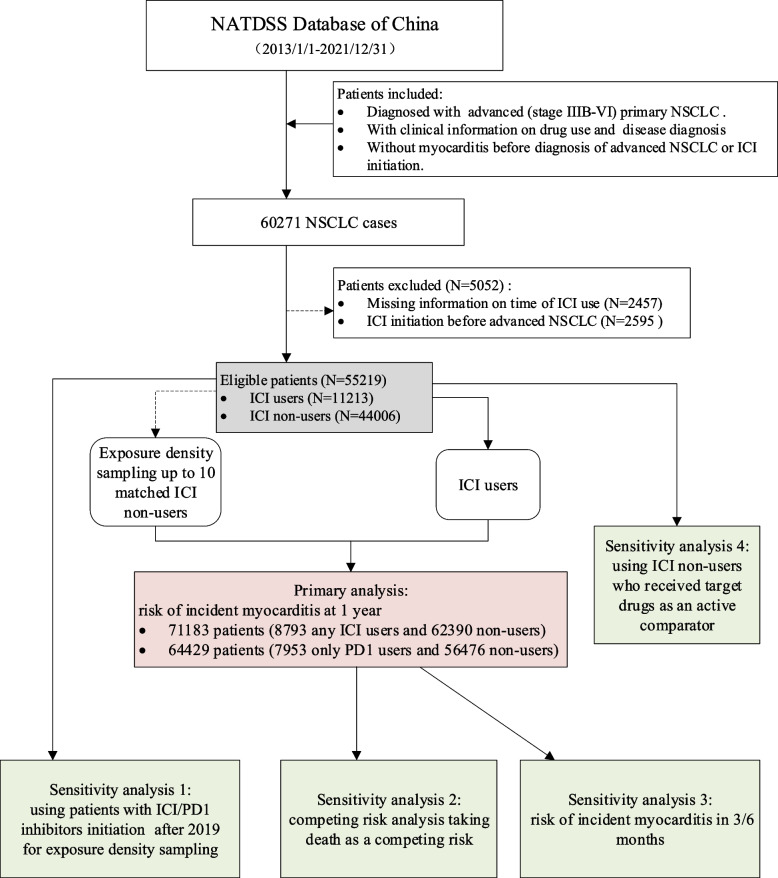
Flowchart of participants from NATDSS Database included in the study. Abbreviation: NATDSS, national anti-tumor drug surveillance system; ICI, immune checkpoint inhibitor; PD1, programmed cell death protein 1; NSCLC, non-small cell lung cancer. The number of ICI non-users in the primary analyses is larger than the initial 55,219 because controls are selected with replacement, therefore one individual can be selected more than once as a control for different ICI users according to the exposure density sampling method

We examined real-world patient data from the National Anti-Tumor Drug Surveillance System (NATDSS) covering more than 10 million cancer patients from over 1400 hospitals in mainland China [18, 19]. This database was initially established for monitoring the use of anti-tumor drugs in clinical practice of China [20]. Comprehensive clinical data from electronic medical records, hospital information system, laboratory information system, picture archiving and communication system, and pathology information system were mandatorily and routinely retrieved from hospitals for administrative surveillance. Mortality data from the national death registries of the China Centers for Disease Control and Prevention were further linked to this database. Natural language processing and other artificial intelligence aided techniques were applied to obtain structured variables. The Chinese National Lung Cancer Cohort (CNLCC) was based on NATDSS [21]. Detailed descriptions of NATDSS and the CNLCC are in the supplementary material. We included all patients newly diagnosed with advanced (stage IIIB-IV) primary NSCLC from the CNLCC. For each patient, the baseline (time 0) was the date when they were first diagnosed with advanced disease. Patients were excluded if they had a history of myocarditis before baseline, or if the time of ICI initiation was missing or before baseline (Fig. 1). We used a new-user design since observational pharmacoepidemiological studies are prone to prevalent user bias [22], where time-dependent risk can result in the early attrition of those individuals most susceptible to the event and in the follow-up of low-risk individuals. The de-identified source data in our analyses were collected between 01 January 2013 and 31 December 2021. The timeline for measurement was illustrated by a diagram (Supplementary Fig. 1).

### Definition of exposures and controls

In the database 13 ICI drugs were used to treat advanced NSCLC in the study time frame, mostly anti-PD1 drugs (Supplementary Table 1) [23]. The primary exposure was defined as any first-ever prescription of ICI therapy that started after baseline. We also examined the prescription specifically of anti-PD1 drugs alone. Those who had not been prescribed any ICIs at the time of matching were defined as controls (non-users). Considering ICI initiation is a time-dependent exposure, we used the exposure density sampling (EDS) approach to match comparable controls and address immortal time bias [24, 25]. Details of EDS were specified in Supplementary material.

### Ascertainment of outcomes

The primary outcome was incident myocarditis within 1 year since the index date. Cases of myocarditis were identified through searching in the medical records of myocarditis clinically diagnosed by health professionals using the International Classification of Disease, 10th Revision (ICD-10) codes (I40/I41/I51.4) and by reviewing diagnosis texts containing “myocarditis” in Chinese. Case severities were not accurately differentiated due to lack of such data. Death from myocarditis could not be ascertained and therefore were not included as an event, because cause of death was not available in the dataset. However, few such deaths are expected. Participants were followed up from the index date until the onset of myocarditis, death, 1 year after the index date, or the end of study [defined as the date of the latest update of source data (December 31, 2021)], whichever came first.

### Assessments of covariates

We included several potential confounders including sex [26], age [12], family history of cancer, prevalent comorbidities [27], status of distant metastasis and prior anti-cancer treatments [28]. Comorbidities including hypertension, diabetes, chronic obstructive pulmonary disease (COPD), coronary heart disease, myocardial infarction, heart failure, and autoimmunity disease were identified by algorithm-based search on clinical records. (Supplementary Table 2) Metastasic status was assessed according to the diagnosis of a secondary malignancy of bone, brain, pericardial, liver, contralateral lung, and adrenal. History of lung cancer-related surgery (if any) was extracted from surgery records. Prior chemotherapy, targeted therapy, and glucocorticoid use were defined as any exposure to related drugs in a predefined list (Supplementary Table 3) through searching prescriptions in medical records. The anti-cancer therapies for included patients were presented in Supplementary Table 4. All covariates mentioned above were firstly measured at baseline and were then updated by the index date for the matched cohort, as illustrated in Supplementary Fig. 1.

### Main analysis

Baseline characteristics were summarized as mean and standard deviation (SD) for continuous variables and frequency and percentages for categorical variables according to the status of ICI use at baseline and index date, respectively. Given the large study size, we assessed the between-group differences in baseline characteristics using standardized mean difference (SMD) for both continuous variables and categorical variables [29], to avoid small p-values produced by traditional tests even when the differences are relatively small [30]. An SMD > 0.1 indicated a between-group imbalance of baseline characteristics.

It has been demonstrated that data obtained from exposure density sampling should be analyzed in the same way as a left-truncated deduplicated cohort rather than a stratified analysis (where each matched pair is a stratum) [24]. Multivariable standard Cox proportional hazard models with time-dependent exposure were used to estimate the hazard ratios (HR) and 95% confidence intervals (CI) for associations between ICI/PD1 inhibitor use and 1-year risk of incident myocarditis. To adjust for age, we used age as the time scale and stratified by birth cohort (every 10-year interval) [31]. The proportional hazards assumption was not violated using Schoenfeld residuals. The 1-year survival rate was calculated by the adjusted Kaplan–Meier method. The E value was calculated to quantify the impact of unmeasured confounding in this observational study [32].

Three models were constructed. The basic model (model 1) adjusted for gender, age at index date, calendar year of index date, and family history. Model 2 additionally adjusted for prevalent comorbidities and metastasis statuses measured at index date on the basis of model 1. The full model (model 3) additionally adjusted for prior treatment interventions measured at index date on the basis of model 2. The main analysis was complete-case analysis and participants with missing values were excluded from the analysis.

### Secondary analysis

To exploratorily examine potential interactions, we conducted subgroup analyses based on gender, age, calendar year, bone metastasis, contralateral lung metastasis, chemotherapy, and glucocorticoid use. These analyses were performed using the full model, focusing on risk factors with potential modification effects while ensuring the number of myocarditis events and sample size allow for the exploratory analysis [12, 26–28]. Potential effect modifications were examined by including the cross-product of the stratifying covariate and exposure into the full model. Evidence of interaction was assessed by using likelihood ratio test comparing models with and without the interaction term.

Several sensitivity analyses were performed using the full model to test the robustness of our main results. First, we excluded ICI users initiated before 2019 because most ICI drugs were approved in China since June 2018 [23]. Subjects with baseline earlier than 2019 were also excluded before the EDS to avoid matching controls with an index date earlier than 2019 and reduce potential bias introduced by calendar time. Second, we performed the Fine-Gray’s subdistribution hazard competing risk regression treating death as a competing event for myocarditis [33]. Third, according to previous reports, myocarditis had a median onset time of 34 days and 81% presenting within 3 months [27], we restricted the length of follow-up to 3 or 6 months to investigate hazard ratios within a shorter observation window. Fourth, we took ICI non-users who received target therapy as an active comparator to further mitigate channeling bias since patients receiving these two drugs shared more similarities in the course of the disease [34]. In this scenario, the index date is the date of initiation for ICI therapy or target therapy.

We used R V.3.6.2 (R Development Core Team) and the packages “survival”, “Epi”, “cmprsk”, “E Value”, and “kmi” for analyses. A *P* < 0.05 (two-sided) was considered statistically significant. This study conforms to the RECORD-PE reporting guidelines [35].

## Results

### Characteristics for eligible patients

Of 60,271 advanced primary NSCLC patients identified in the NATDSS database, 55,219 (median age at diagnosis, 61 years; male, 62%) were eligible for the analyses (Table 1). They came from 22 provincial-level regions (covering 69% of all 32 regions in mainland China) (Fig. 1). Among these eligible patients, there were 33 cases of myocarditis among 11,213 who had started ICI therapy after diagnosis and 55 cases among 44,006 who had never used ICI therapies during the study period. The median onset time of myocarditis since ICI initiation was 59 days and 64% presented within 3 months (the corresponding figures among cases of myocarditis since baseline among ICI non-users were 106 days and 45%). ICI users were more likely to be male and have adrenal metastasis, and less likely to have brain metastasis (Table 1). ICI users also appeared to have a family history of cancer. However, apart from gender, the magnitude of these baseline differences may not be clinically significant.

**Table 1:** Baseline characteristics of all eligible participants and the cohort matched on exposure density

	Eligible participants (*N* = 55,219)			Matched cohort^a^ (*N* = 71,183)		
	ICI users N (%)	ICI non-users N (%)	SMD	ICI users N (%)	Matched Controls N (%)	SMD^b^
Total	11213	44006		8793	62390	
Gender (male)	8374 (74.7%)	26,125 (59.4%)	0.330	6646 (75.6%)	48,025 (77.0%)	0.033
Age at baseline						
Mean (SD)	60 (9.98)	60 (10.40)	0.034	60 (9.22)	61 (8.56)	0.023
Median (quartile)	61 (54, 67)	61 (53, 67)		61 (55,67)	61 (55, 67)	
Birth year						
< 1950	1579 (14.1%)	7418 (16.9%)	0.088	1057 (12.0%)	8584 (13.8%)	0.126
1950–1960	4496 (40.1%)	16,551 (37.6%)		3655 (41.6%)	27,561 (44.2%)	
1960–1970	3760 (33.5%)	14,227 (32.3%)		3087 (35.1%)	21,268 (34.1%)	
> 1970	1378 (12.3%)	5810 (13.2%)		994 (11.3%)	4977 (8.0%)	
Family history of cancer						
Yes	808 (7.2%)	2291 (5.2%)	0.292	624 (7.1%)	3428 (5.5%)	0.283
Missing	748 (6.7%)	6854 (15.6%)		622 (7.1%)	9935 (15.9%)	
Bone metastasis	4249 (37.9%)	18,484 (42.0%)	0.084	3323 (37.8%)	24,055 (38.6%)	0.016
Brain metastasis	2350 (21.0%)	11,522 (26.2%)	0.123	1735 (19.7%)	11,895 (19.1%)	0.017
Pericardial metastasis	90 (0.8%)	325 (0.7%)	0.007	20 (0.2%)	27 (0.0%)	0.050
Liver metastasis	1756 (15.7%)	6462 (14.7%)	0.027	1166 (13.3%)	6776 (10.9%)	0.074
Contralateral Lung metastasis	4001 (35.7%)	14,877 (33.8%)	0.039	3090 (35.1%)	21,765 (34.9%)	0.005
Adrenal metastasis	1074 (9.6%)	2852 (6.5%)	0.114	533 (6.1%)	2335 (3.7%)	0.108
Hypertension	2002 (17.9%)	6929 (15.7%)	0.056	1146 (13.0%)	5022 (8.0%)	0.163
Diabetes	1001 (8.9%)	3040 (6.9%)	0.075	439 (5.0%)	1149 (1.8%)	0.174
COPD	287 (2.6%)	919 (2.1%)	0.031	82 (0.9%)	178 (0.3%)	0.083
Coronary heart disease	97 (0.9%)	309 (0.7%)	0.019	3 (0.0%)	3 (0.0%)	0.021
Myocardial infarction	49 (0.4%)	101 (0.2%)	0.036	2 (0.0%)	2 (0.0%)	0.017
Heart failure	78 (0.7%)	408 (0.9%)	0.026	6 (0.1%)	7 (0.0%)	0.029
Autoimmunity disease	74 (0.7%)	431 (1.0%)	0.035	7 (0.1%)	9 (0.0%)	0.030
Surgery	776 (6.9%)	3283 (7.5%)	0.021	334 (3.8%)	796 (1.3%)	0.161
Chemotherapy	3086 (27.5%)	11,257 (25.6%)	0.044	2023 (23.0%)	11,097 (17.8%)	0.130
Targeted therapy	566 (5.0%)	1914 (4.3%)	0.033	197 (2.2%)	388 (0.6%)	0.137
Glucocorticoid	3508 (31.3%)	12,915 (29.3%)	0.042	2332 (26.5%)	12,838 (20.6%)	0.140
Days from baseline to ICI initiation						
Median (IQ)	44 (8, 229)	——		40 (8, 209)	——	
Mean (SD)	189 (324)	——		168 (280)	——	
ICI scheme						
PD1 inhibitors only	10,008 (89.3%)	——		7856 (89.3%)	——	
PDL1 inhibitors only	1042 (9.3%)	——		813 (9.2%)	——	
PDl&PDL1 inhibitors	121 (1.1%)	——		89 (1.0%)	——	
CTLA-4 (with other ICI or not)	42 (0.4%)	——		35 (0.4%)	——	

*Abbreviation*: *ICI* immune checkpoint inhibitor, *SD* standard deviation, *COPD* chronic obstructive pulmonary disease, *PD1* programmed cell death protein 1, *PDL1* programmed cell death ligand 1, *CTLA-4* Cytotoxic T lymphocyte antigen 4

^a^up to 10 controls were matched for each ICI user using an “exposure density sampling” approach

^b^SMD, standardized mean difference (shown as an absolute value). An SMD > 0.1 indicated a between-group imbalance of baseline characteristics

### Characteristics for the analysis cohorts

In the cohort for analysis, ICI users (71,183 patients; median age at diagnosis, 61 years; male, 77%) and PD1 inhibitor users (64,429 patients; median age at diagnosis, 61 years; male, 76%) were more likely to have adrenal metastasis, hypertension, diabetes and prior cancer treatment at baseline (Table 1 and Supplementary Table 5), and likewise when measured at the index date (Supplementary Table 6). When compared with the active comparator of patients receiving target therapy, ICI users were more likely to have diabetes, prior chemotherapy, and metastasis of brain, contralateral lung, and adrenal at baseline (Supplementary Table 7).

### Myocarditis in the matched cohort

In the matched cohort for any ICI user, there were 54 patients who developed myocarditis within the first year, over a median follow-up of 335 days since the index date (231 days for ICI users and 358 days for non-users) (Table 2). The cumulative incidence of myocarditis was 4.8 per 1000 person-years among ICI users (26 events in 8793 patients) compared to 0.6 per 1000 person-years, among ICI non-users (28 events in 62,390 patients). These unadjusted estimates represent an absolute risk difference of 4.2 per 1000 person-years. The adjusted Kaplan–Meier curves are in Supplementary Fig. 2. The median time from the index date to onset of myocarditis was 47.5 and 166.5 days in the ICI users and non-users respectively.

**Table 2:** Association between ICI initiation and risk of incident myocarditis within 1 year in advanced NSCLC patients of China

ICI scheme	Exposure		Control		Hazard ratio (95% confidence interval)		
	Participants N	Events N (%)	Participants N	Events N (%)	model 1^a^	model 2^b^	model 3^c^
Any ICI	8793	26 (0.30)	62,390	28 (0.04)	7.78 (3.51–17.26)***	7.40 (3.33–16.48)***	7.41 (3.29–16.67)***
PD1 inhibitors	7953	25 (0.31)	56,476	27 (0.05)	8.85 (3.88–20.20)***	8.43 (3.66–19.42)***	8.39 (3.56–19.77)***

*Abbreviation*: *ICI* immune checkpoint inhibitor, *NSCLC* non-small cell lung cancer, *N* number, *PD1* programmed cell death protein 1

^a^Model 1 was adjusted for gender, index age, year of the index date, and family history

^b^Model 2 was additionally adjusted for comorbidities and metastasis conditions by index date on the basis of model 1

^c^Model 3 was additionally adjusted for prior treatment interventions by index date on the basis of model 2

^***^ *p* value < 0.001

In the sub cohort for PD1 inhibitor users, there were 52 patients who developed myocarditis within the first year. The cumulative incidence of myocarditis was 5.1 per 1000 person-years among PD1 inhibitor users (25 events in 7953 patients) compared to 0.7 per 1000 person-years, among matched ICI non-users (27 events in 56,476 patients). The median time from the index date to onset of myocarditis was 47 and 157 days in the PD1 inhibitor users and non-users respectively.

### Association of ICI use with myocarditis

Compared with model 1 simply adjusted for gender, index age, index year and family history, progressive adjustment for comorbidities, metastasis and prior treatment interventions by the index date (model 2 and model 3) slightly weakened the effect estimates (Table 2). In the fully adjusted model (model 3), ICI initiation was associated with an increase in the risk of myocarditis compared with no ICI initiation (HR = 7.41, 95% CI 3.29 ~ 16.67, *P* < 0.001). Similarly, PD1 inhibitor initiation was associated with an increased risk (HR = 8.39, 95% CI 3.56 ~ 19.77, *P* < 0.001) in the full model. Although the confidence intervals were wide, even the lower limits showed a threefold increased risk. However, these relative risks should be interpreted alongside the small absolute risk difference. The E values for the hazard ratio of 7.41 and 8.39 was estimated to be 14.3 and 16.3, respectively.

### Subgroup analysis

**Fig. 2:**
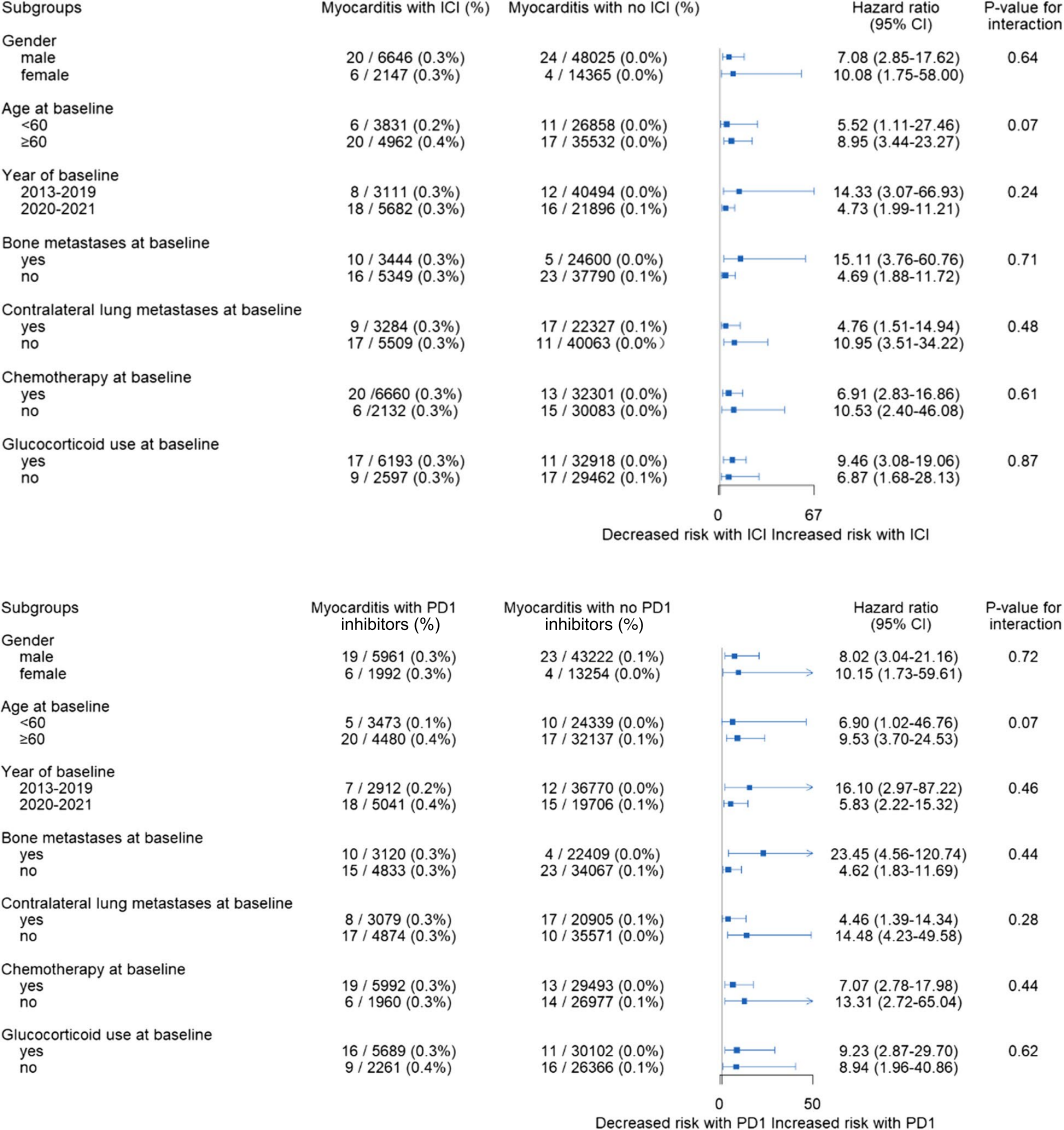
Subgroup analyses. The association between ICI/PD1 inhibitors initiation and risk of incident myocarditis within 1 year of follow-up in advanced NSCLC patients of China

Although the number of patients who developed myocarditis is relatively small in each of the subgroups examined, the hazard ratios indicated an increased risk across all subgroup factors (Fig. 2). There was a suggestion that the effect might be greater in ICI users aged at least 60 years at baseline, compared to < 60 years (HR 8.95 vs 5.52, *p* = 0.07 among any ICI users; HR 9.53 vs 6.90, *p* = 0.07 among PD1 inhibitor users). No differences were observed between other pre-specified subgroups for ICI use or PD1 inhibitor use.

### Sensitivity analysis

The sensitivity analyses produced consistent findings (Table 3). First, the fully adjusted HR changed in the any ICI cohort (from 7.41 to 5.22) and the PD1 cohort (from 8.39 to 5.86) when death was analyzed as a competing event for myocarditis. Second, the effect remained similar (HRs 7.59 and 8.18) when the follow-up period was restricted to 3 months and increased to 10.93 and 11.63 when restricted to 6 months. Third, when using target therapies as an active comparator, the hazard ratios were still high (7.29 for any ICI and 7.83 for PD1 inhibitors only).

**Table 3:** Five Sensitivity analyses for the association between ICI use and risk of incident myocarditis in advanced NSCLC patients of China

ICI scheme	Hazard ratio (95% confidence interval)^a^				
	Excluding ICI users initiated before 2019	Competing risk analysis^b^	Risk within 6 months	Risk within 3 months	Using target therapy as an active comparator
Any ICI	7.44 (3.28–16.86)***	5.22 (2.53–10.80)***	10.93 (4.29–27.83)***	7.59 (2.75–20.93)***	7.29 (2.12–25.07)**
PD1 inhibitors	8.30 (3.51–19.66)***	5.86 (2.72–12.60)***	11.63 (4.56–29.68)***	8.18 (2.98–22.49)***	7.83 (2.27–27.04)***

*Abbreviation: ICI* immune checkpoint inhibitor, *NSCLC* non-small cell lung cancer, *N* number, *PD1* programmed cell death protein 1

^a^All sensitivity analyses were performed using the full model adjusted for gender, index age, year of the index date, family history, comorbidities, metastasis conditions, and prior treatment interventions

^b^death as a competing risk

^**^p value < 0.01

*** *p* value < 0.001

## Discussion

### Summary of principal findings

In this large nationwide cohort study we found that ICI initiation in advanced NSCLC was significantly associated with a sevenfold increase in the 1-year risk of myocarditis after adjustment for gender, age, year of the index date, family history, prevalent comorbidities, metastasis statuses, and prior treatment interventions. No significant interactions were observed. Results from sensitivity analyses agreed with our main findings of increased myocarditis risk following ICI initiation.

### Comparisons with other studies

Two systematic reviews have respectively reported contradictory results. One study found an non-significant association (RR = 1.11, 95% CI: 0.64–1.92) with 17 myocarditis [14], which provoked debate in the context of numerous evidence indicating a potential association [36]. In response, the later one excluded trials with no events of myocarditis in either ICI user and non-user arms [37], and reported a significant association (Peto OR = 4.42, 95% CI: 1.56–12.50) with 15 myocarditis using refined statistical methods [15]. Compared with our study (HR = 7.41, 95% CI: 3.29–16.67), association from systematic reviews seemed weaker, probably because of insufficient length of follow-up in some trials included (only up to 3 months). In addition, clinical trials often have highly selective study populations and small sample sizes [38], making it difficult to examine rare adverse events even when the trials are pooled [39]. As observed in our study, the average HR within 6 months was higher than that within 3 months, suggesting a potential increase in risk beyond the early period. This elevated HR beyond 3 months may reflect the delayed onset of ICI-related myocarditis in real-world settings. Notably, previous clinical reports have primarily documented fatal and fulminant cases occurring within the first 3 months, while milder cases with a delayed onset may have contributed to the increased HR observed beyond this timeframe. However, since our study lacked data on myocarditis severity, further investigation is warranted to confirm this possibility. On the other hand, previous estimates from case–control studies based on the VigiBase (OR = 9.66, 95% CI: 7.16–13.05) and FAERS database (ROR = 11.21, 95% CI: 9.36–13.43) [6, 12] seemed appreciably larger than ours. These studies used comparators that consisted of all drugs (for estimating ROR) or other non-ICI drugs (for estimating OR) in the database taken by patients diagnosed with different diseases. Such comparators were less likely to induce myocarditis than those non-ICI cardio-risking cancer treatments included in our study, and thus resulted in larger estimates of relative risk.

Only one relevant cohort study was conducted among 25,573 lung cancer patients in Denmark and reported an increased 1-year risk of a composite endpoint of peri- or myocarditis (only 11 outcome events, HR = 5.51, 95% CI: 2.85–10.66) after adjustment for age, gender, and time since diagnosis [13] This is comparable to our findings. Due to the existence of immortal time, “a period of follow-up during which, by study design, death or the outcome cannot occur before exposure”, susceptible patients who seemed to have higher risk developed myocarditis shortly after cancer diagnosis and were directly classified into the control group in the Danish study [40].

### Possible mechanisms

Myocarditis, regardless of etiologies, always begins with an immune-mediated injury to the myocardium [41]. Cardiomyocytes activate CTLA-4 and PD-1/PD-L1 pathways to limit T cell response under physiological conditions. The application of ICIs blocks these pathways, thus over-activating T cells and disturbing immunologic homeostasis in the heart [42]. In the early case report of ICI-associated myocarditis, histological analysis in humans confirmed infiltration of T lymphocytes (both CD4 and CD8 cells) and macrophages, suggesting immune infiltration as the main pathophysiological driver [43]. A recent study revealed that autoreactive T cells against cardiac antigens may contribute to ICI–related myocarditis [44].

### Strengths and limitations

Compared with previous studies, this research benefits from the largest sample size of NSCLC patients and also the largest number of myocarditis events to date with enriched clinical information and follow-up data, which enabled rigorous pharmacoepidemiology study design and several secondary analyses (showing consistensy) for the investigation of rare outcomes. Real-world nationwide patient data also ensured good representativeness of clinical practice settings. Given that heterogeneities of ICI-related cardiovascular toxicity were observed across different cancer types and stages [45], selecting patients of the same cancer type and stage could help reduce selection bias between groups [34]. We adopted a new-user design (excluding prevalent ICI initiators) to avoid prevalent-user bias. Besides, we applied the exposure density sampling, an improved approach to mitigate potential immortal time bias introduced by time-dependent exposures in observational studies.

There are several limitations in this study. First, we only adopted cases clinically diagnosed by health professionals and did not additionally define outcomes through existing clinical examinations and laboratory tests. Nevertheless, we believed that health professionals made diagnosis of myocarditis for cancer patients aligning well with clinical guidelines. We could not accurately differentiate disease severities and assess myocarditis-related deaths due to lack of available data. Besides, information bias such as misdiagnosis of myocarditis due to insufficient identification might exist as practical guidelines for clinical surveillance and standard diagnosis of ICI-related cardio-toxities were not available until the June of 2018 [46], which could result in underestimation of the risk of ICI-related myocarditis. However, when we excluded ICI initiators and matched controls before 2019 in sensitivity analysis, the results were similar to our main findings. Second, it has been reported that combined therapy of CTLA-4 and PD1/PDL1 inhibitors are more likely to induce myocarditis compared with monotherapy of PD1/PDL1 inhibitors [6, 10, 12, 27, 47]. Risk of myocarditis by different ICI schemes seemed diverse and remained to be uncovered. We are unable to investigate association between different ICI schemes and myocarditis due to limited number of patients using CTLA-4 (*n* = 42). Nevertheless, a proportion of 90% included patients in this study initiated PD1 inhibitors, and our findings could still be of major clinical relevance if the proportion corresponds with real-world ICI utilization. Third, this study might be susceptible to indication bias since patients without EGFR or ALK mutations are not recommended for ICI [3] and these data were not available in the NATDSS database. However, in the sensitivity analysis using initiators of target drugs as an active comparator, the association between ICIs and myocarditis remained significant despite wider confidence intervals due to smaller sample size and fewer events. Similarly, other unmeasured confounding, such as radiotherapy, may still exist although we had tried to reduce the impact of potential confounders on our estimates. Nevertheless, according to the E value estimation, the unmeasured confounding must be associated with both ICI/PD1 inhibitor initiation and risk of myocarditis by a HR of 14.3/16.3 to fully explain away the observed HR of 7.41/8.39. Fourth, given the low incidence of this rare outcome, our subgroup analysis may lack sufficient statistical power. Further investigations are needed to explore this potential interaction more robustly.

### Clinical and research implications

In accordance with previous studies, our study found notably increased risk of myocarditis induced by ICI initiation in advanced NSCLC patients. Furthermore, we observed a longer median onset time of myocarditis following ICI initiation (59 days) compared to 34 days and a larger proportion of myocarditis occurred beyond 3 months (36% vs 19%) in previous reports [27]. Also, the average hazard ratio for 6 months was higher than that for 3 months (10.93 vs 7.59) in our study. These highlight a longer duration of surveillance for myocarditis after ICI initiation should be warranted.

Further studies are needed to clarify whether the association of ICI therapy with myocarditis is seen in other cancer sites, and whether type and dose of ICIs matter. Further investigation is warranted to confirm whether the risk of ICI-associated myocarditis remains elevated beyond 3 months up to 6 months and, if so, to elucidate the underlying reasons for this increased risk. Laboratory indicators or molecular markers could be developed to help diagnose myocarditis early in susceptible people.

## Conclusion

ICI therapies in advanced NSCLC patients are associated with a sevenfold increased risk of myocarditis within one year following initiation, which suggests close monitoring for myocarditis especially within 6 months for patients considered susceptible, acknowledging that the event rate and the absolute risk is relatively small. Further studies are needed to confirm whether the risk varies by type of ICI, and if there is a dose–response relationship.

## Supplementary Information

Supplementary Material 1.

Supplementary Material 2.

## Data Availability

All data analyzed in this study were provided by the National Cancer Center of China under approval of research proposal and are available on reasonable request, subject to permission by the National Cancer Center of China.

## Abbreviations

EDS: Exposure density sampling
ICI: Immune checkpoint inhibitor
NATDSS: National Anti-Tumor Drug Surveillance System
NSCLC: Non-small cell lung cancer
PD 1: Programmed cell death protein 1
PDL1: Programmed cell death ligand 1
