# Supplementary material for: Immune checkpoint inhibitors and myocarditis in advanced non-small cell lung cancer: a nationwide cohort study
Source: Cardiooncology. 2025 Mar 31;11:33. doi: 10.1186/s40959-025-00325-6 (PMC11956456; doi:10.1186/s40959-025-00325-6)
Supplement: Supplementary file 2 — Supplementary Material 2. [file 40959_2025_325_MOESM2_ESM.docx]

**The NATDSS database**

NATDSS is established by the National Cancer Center for monitoring the use of anti-tumor drugs in clinical practice at a national level. NATDSS is a representative database with demographically and geographically diverse information covering more than 10 million cancer patients from over 1400 hospitals in mainland China, including cancer hospitals and tier-three general hospitals with oncology departments. Clinical data from the Electronic Medical Record, Hospital Information System, Laboratory Information Management System, Picture Archiving and Communication System, and Pathology Information System were routinely retrieved to collect information on demographic characteristics, drug prescriptions, diagnoses, examinations, laboratory tests, treatments, surgery records, and follow-up. Based on the NATDSS database, the Chinese National Lung Cancer Cohort (CNLCC) was launched by the National Cancer Center, the Cancer Institute and Hospital, and the Chinese Academy of Medical Sciences, in collaboration with institution leaders, senior thoracic surgeons, oncologists, local principal investigators, pathologists and biostatisticians. The program retrieves mortality data from the national death registries of the China Centers for Disease Control and Prevention (CDC). Algorithm of natural language processing and other artificial intelligence aided techniques were applied to obtain structured variables.

**The exposure density sampling (EDS) approach**

EDS performs dynamic matching for exposures occurring over time, in contrast to outcome-dependent matching applied in a nested case-control study. For each exposed individual, i.e. ICI initiator, we matched up to 10 controls without replacement at the time of exposure from the risk set, namely those who were at risk for an event (myocarditis/death) and still non-exposed at that time. Except for time to exposure, we also matched for gender and other baseline characteristics including age, comorbidities, status of metastasis, and prior treatment interventions (specified below). The index date, namely the start of follow-up, was defined as the time of exposure for ICI users and the time of matching for controls. Controls for different ICI users were sampled from the risk sets with replacement to derive unbiased estimators. One individual could serve as a control for several ICI users and hence the total number of controls in the primary analysis is larger than the initial ICI non-users included. In that case, the earliest matching date was considered the index date for that individual. Besides, if a (as-yet unexposed) control was successfully matched several times and became exposed later, the index date would still be the earliest matching time, meanwhile, the exposure status was included as a time-dependent variable for this individual. A separate subset was created using patients who had PD1 drugs alone as exposed patients and their matched non-users as controls.

**Supplementary Figure 1** Study design diagram with measurement of exposures, outcomes, and covariates.

* Index date is the start of at-risk time for Cox regression and defined as the date of ICI initiation for ICI users and the date of matching for controls, ranging from baseline and the end of study (2021/12/31).

^a^ Covariates used for matching in the exposure density sampling included gender, baseline age, and health conditions and intervention (prior treatment /comorbidities/metastasis) measured at baseline.

^b^ Covariates adjusted in the Cox regression included gender, index age, and health conditions and intervention (prior treatment /comorbidities/metastasis) measured by index date.

Abbreviation: ICI, Immune-Checkpoint-Inhibitor; NSCLC, non-small cell lung cancer.

**
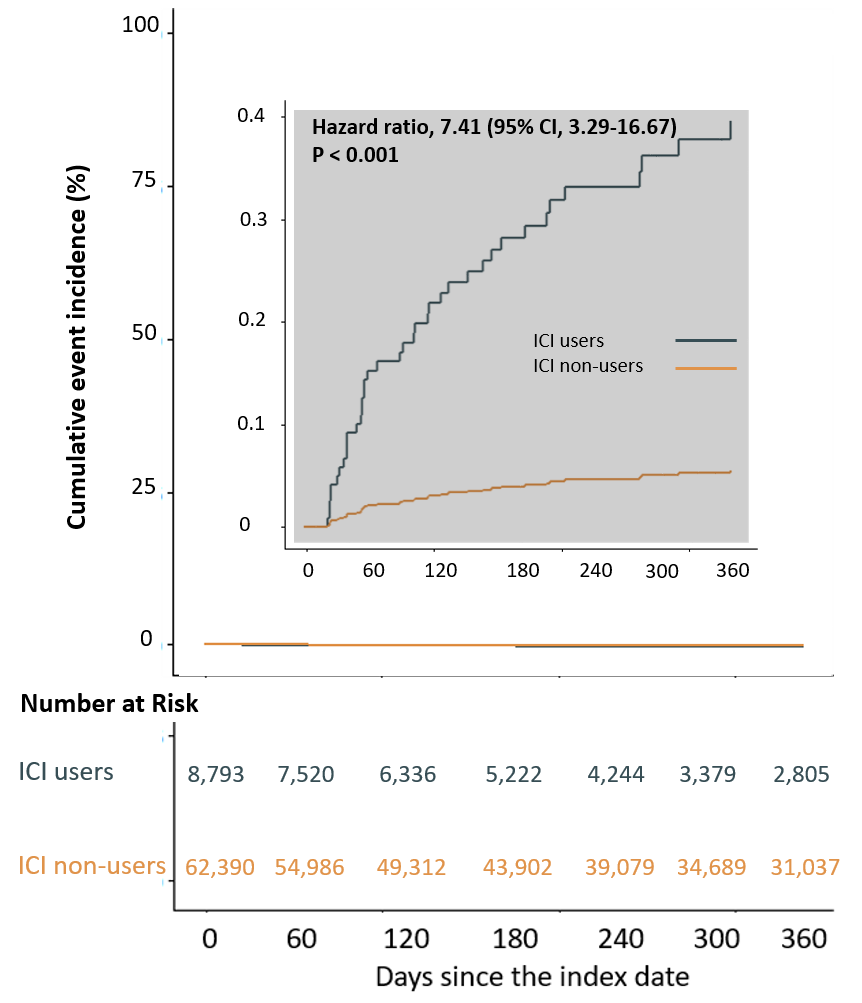
**

**Supplementary Figure 2** Kaplan-Meier curves for the 1-year cumulative event incidence.

* Index date is the start of at-risk time for Cox regression and is defined as the date of ICI initiation for ICI users and the date of matching for non-users.

Abbreviation: ICI, Immune-Checkpoint-Inhibitor

**Supplementary Table 1. Detailed information on ICI use for 55219 eligible participants.**

| **ICI** | **Drugs** | | **Number of patients (%)** | |  |
| --- | --- | --- | --- | --- | --- |
| **PD1** | | **Nivolumab** | | **724 (1.3%)** | |
|  |  | **Pembrolizumab** | | **1973 (3.6%)** | |
|  |  | **Camrelizumab** | | **2872 (5.2%)** | |
|  |  | **Toripalimab** | | **968 (1.8%)** | |
|  |  | **Sintilimab** | | **3295 (6.0%)** | |
|  |  | **Tislelizumab** | | **1493 (2.7%)** | |
|  |  | **Penpulimab** | | **48 (0.1%)** | |
|  |  | **Zimberelimab** | | **8 (0.0%)** | |
|  |  | **Geptanolimab** | | **8 (0.0%)** | |
| **PDL1** | | **Durvalumab** | | **654 (1.2%)** | |
|  |  | **Atezolizumab** | | **586 (1.1%)** | |
|  |  | **Avelumab** | | **0 (0.0%)** | |
| **CTLA-4** | | **Ipilimumab** | | **42 (0.1%)** | |

**Supplementary Table 2. Description of comorbidities in the present study.**

| ***Comorbidities*** | ***Measurement*** |
| --- | --- |
| **Hypertension** | **The first diagnosis was identified through an algorithm-based search of clinical records with information of ICD codes (I10-I15) and related text.** |
| **Diabetes** | **The first diagnosis was identified through an algorithm-based search of clinical records with information of ICD codes (E10-E14) and related text.** |
| **COPD** | **The first diagnosis was identified through an algorithm-based search of clinical records with information of ICD codes (J44) and related text.** |
| **Coronary heart disease** | **The first diagnosis was identified through an algorithm-based search of clinical records with information of ICD codes (I25) and related text.** |
| **Myocardial infarction** | **The first diagnosis was identified through an algorithm-based search of clinical records with information of ICD codes (I21/I22/I23/I24) and related text.** |
| **Heart failure** | **The first diagnosis was identified through an algorithm-based search of clinical records with information of ICD codes (I50) and related text.** |
| **Autoimmunity disease** | **The first diagnosis was identified through an algorithm-based search of clinical records with information of ICD codes (I00, M06.8, M06.9, L40, M32, and K50-K52) and related text.** |

**Supplementary Table 3. Drug list for chemotherapy, target therapy, and glucocorticoid.**

| **Treatment** | **Drugs** |
| --- | --- |
| **Chemotherapy** | **Recombinant human vascular endostatin** |
|  | **Everolimus** |
|  | **Vinorelbine** |
|  | **Vincristine** |
|  | **Paclitaxel** |
|  | **Loboplatin** |
|  | **Cisplatin** |
|  | **Carboplatin** |
|  | **Nidaplatin** |
|  | **Pemetrexed** |
|  | **Docetaxel** |
|  | **Gemcitabine** |
|  | **Capecitabine** |
|  | **Irinotecan** |
|  | **Topotecan** |
|  | **Etoposide** |
|  | **Cyclophosphamide** |
|  | **Nimustine** |
|  | **Nitrokamustard** |
|  | **Ifosfamide** |
|  | **Hexamethylmelamine** |
|  | **Methotrex** |
|  | **Epirubixin** |
|  | **Bleomycin** |
|  | **Doxorubicin** |
|  | **Doxorubicin liposomes** |
|  | **Mitomycin** |
|  | **Sodium spotatoate** |
|  | **Elemicene** |
|  | **Vindesine** |
| **Target therapy** | **Osimertinib** |
|  | **Gefitinib** |
|  | **Erlotinib** |
|  | **Eclitinib** |
|  | **Afatinib** |
|  | **Dacotinib** |
|  | **Ametinib** |
|  | **Eflutinib** |
|  | **Anlotinib** |
|  | **Crizotinib** |
|  | **Savolitinib** |
|  | **Beretitinib** |
|  | **Ceritinib** |
|  | **Alectinib** |
|  | **Bevacizumab** |
|  | **Omalizumab** |
|  | **Ointozumab** |
|  | **Bariximab** |
|  | **Denosumab** |
|  | **Emmetrituzumab** |
|  | **Ramolurumab** |
|  | **Ranibizumab** |
|  | **Rituximab** |
|  | **Nexituzumab** |
|  | **Nilotuzumab** |
|  | **Palivizumab** |
|  | **Pertuzumab** |
|  | **Trastuzumab** |
|  | **Tocilizumab** |
|  | **Vedolizumab** |
|  | **Vedicitumab** |
|  | **Cetuximab** |
|  | **Enitumab** |
| **Glucocorticoid** | **Hydrocortisone** |
|  | **Prednisolone** |
|  | **Prednisone** |
|  | **cortisone** |
|  | **Halogmethasone** |
|  | **Methylprednisone** |
|  | **Mometasone furoate** |
|  | **dexamethasone** |
|  | **Betamethasone** |
|  | **Methylprednisolone** |

**Supplementary Table 4. Anti-cancer drug therapy for 55219 eligible participants.**

| **Anti-cancer therapy** | **Number of patients (%)** |
| --- | --- |
| Single agent ICI only | 1351 (2.4%) |
| ICIs only | 112 (0.2%) |
| Chemotherapy only | 23781 (43.1%) |
| Targeted therapy only | 2403 (4.4%) |
| Combination of chemotherapy plus targeted therapy | 7123 (12.9%) |
| Combination of ICI plus targeted therapy | 451 (0.8%) |
| Combination of ICI plus chemotherapy | 6268 (11.4%) |
| Combination of ICI plus chemotherapy plus targeted therapy | 3031 (5.5%) |
| None of the above | 10699 (19.4%) |

**Supplementary Table 5** Baseline characteristics for the matched cohort with the exposure group defined by users of PD1 (N=64429).

|  | PD1 users  N (%) | Matched Controls ^a^  N (%) | SMD ^b^ |
| --- | --- | --- | --- |
| Total | 7953 | 56476 |  |
| Gender (male) | 5961 (75.0%) | 43222 (76.5%) | 0.037 |
| Age at baseline |  |  |  |
| Mean (SD) | 60 (9.27) | 61 (8.60) | 0.026 |
| Median (quartile) | 61 (55, 67) | 61 (55, 67) |  |
| Birth year |  |  |  |
| <1950 | 972 (12.2%) | 7945 (14.1%) | 0.126 |
| 1950-1960 | 3280 (41.2%) | 24,751 (43.8%) |  |
| 1960-1970 | 2812 (35.4%) | 19,315 (34.2%) |  |
| >1970 | 889 (11.2%) | 4465 (7.9%) |  |
| Family history of cancer |  |  |  |
| Yes | 547 (6.9%) | 3106 (5.5%) | 0.280 |
| Missing | 572 (7.2%) | 9038 (16.0%) |  |
| Bone metastasis | 3014 (37.9%) | 21,912 (38.8%) | 0.019 |
| Brain metastasis | 1510 (19.0%) | 10,388 (18.4%) | 0.015 |
| Pericardial metastasis | 20 (0.3%) | 27 (0.0%) | 0.053 |
| Liver metastasis | 976 (12.3%) | 5592 (9.9%) | 0.076 |
| Contralateral Lung metastasis | 2897 (36.4%) | 20,391 (36.1%) | 0.007 |
| Adrenal metastasis | 453 (5.7%) | 1988 (3.5%) | 0.104 |
| High blood pressure | 1009 (12.7%) | 4437 (7.9%) | 0.160 |
| Diabetes | 380 (4.8%) | 990 (1.8%) | 0.171 |
| COPD | 70 (0.9%) | 157 (0.3%) | 0.079 |
| Coronary heart disease | 3 (0.0%) | 3 (0.0%) | 0.022 |
| Myocardial infarction | 1 (0.0%) | 1 (0.0%) | 0.013 |
| Heart failure | 6 (0.1%) | 7 (0.0%) | 0.030 |
| Autoimmunity disease | 3 (0.0%) | 3 (0.0%) | 0.022 |
| Surgery | 318 (4.0%) | 763 (1.4%) | 0.165 |
| Chemotherapy | 1872 (23.5%) | 10,288 (18.2%) | 0.131 |
| Targeted therapy | 188 (2.4%) | 377 (0.7%) | 0.139 |
| Glucocorticoid | 2170 (27.3%) | 11,924 (21.1%) | 0.144 |
| Days from baseline to PD1 initiation | |  |  |
| Median (quartile) | 111 (19, 363) | —— |  |
| Mean (SD) | 268 (383) | —— |  |

^a^ up to 10 controls were matched for each ICI user using an “exposure density sampling” approach.

^b^ SMD, standardized mean difference (shown as an absolute value). An SMD >0.1 indicated a between-group imbalance of baseline characteristics.

Abbreviation: SD, standard deviation; COPD, chronic obstructive pulmonary disease; PD1, programmed cell death receptor-1.

**Supplementary Table 6** Characteristics at index date for the matched cohort with the exposure group defined by users of any ICI, and PD1.

|  | ICI users  N (%) | Matched  Controls ^a^ N (%) | SMD ^b^ | PD-1 users  N (%) | Matched  Controls N (%) | SMD |
| --- | --- | --- | --- | --- | --- | --- |
| Total | 8793 | 62,390 |  | 7953 | 56,476 |  |
| Gender (male) | 6646 (75.6%) | 48,025 (77.0%) | 0.033 | 5961 (75.0%) | 43,222 (76.5%) | 0.037 |
| Age at index date |  |  |  |  |  |  |
| Mean (SD) | 61 (9.13) | 61 (8.49) | 0.015 | 61 (9.17) | 61 (8.53) |  |
| Median (quartile) | 62 (55, 67) | 62 (55, 67) |  | 62 (55, 67) | 62 (55, 67) | 0.018 |
| Birth year |  |  |  |  |  |  |
| <1950 | 1057 (12.0%) | 8584 (13.8%) | 0.126 | 972 (12.2%) | 7945 (14.1%) | 0.126 |
| 1950-1960 | 3655 (41.6%) | 27,561 (44.2%) |  | 3280 (41.2%) | 24,751 (43.8%) |  |
| 1960-1970 | 3087 (35.1%) | 21,268 (34.1%) |  | 2812 (35.4%) | 19,315 (34.2%) |  |
| >1970 | 994 (11.3%) | 4977 (8.0%) |  | 889 (11.2%) | 4465 (7.9%) |  |
| Family history of cancer |  |  |  |  |  |  |
| Yes | 624 (7.1%) | 3428 (5.5%) | 0.283 | 547 (6.9%) | 3106 (5.5%) | 0.280 |
| Missing | 622 (7.1%) | 9935 (15.9%) |  | 572 (7.2%) | 9038 (16.0%) |  |
| Bone metastasis | 3444 (39.2%) | 24,600 (39.4%) | 0.005 | 3120 (39.2%) | 22,409 (39.7%) | 0.009 |
| Brain metastasis | 1834 (20.9%) | 12,384 (19.8%) | 0.025 | 1598 (20.1%) | 10,837 (19.2%) | 0.023 |
| Pericardial metastasis | 25 (0.3%) | 41 (0.1%) | 0.052 | 24 (0.3%) | 40 (0.1%) | 0.054 |
| Liver metastasis | 1264 (14.4%) | 7105 (11.4%) | 0.089 | 1061 (13.3%) | 5894 (10.4%) | 0.090 |
| Contralateral Lung metastasis | 3284 (37.3%) | 22,327 (35.8%) | 0.032 | 3079 (38.7%) | 20,905 (37.0%) | 0.035 |
| Adrenal metastasis | 616 (7.0%) | 2544 (4.1%) | 0.128 | 530 (6.7%) | 2182 (3.9%) | 0.126 |
| Hypertension | 1279 (14.5%) | 5646 (9.0%) | 0.171 | 1130 (14.2%) | 5017 (8.9%) | 0.167 |
| Diabetes | 516 (5.9%) | 1460 (2.3%) | 0.179 | 452 (5.7%) | 1280 (2.3%) | 0.176 |
| COPD | 104 (1.2%) | 231 (0.4%) | 0.093 | 90 (1.1%) | 204 (0.4%) | 0.090 |
| Coronary heart disease | 13 (0.1%) | 20 (0.0%) | 0.039 | 10 (0.1%) | 18 (0.0%) | 0.033 |
| Myocardial infarction | 4 (0.0%) | 13 (0.0%) | 0.014 | 1 (0.0%) | 10 (0.0%) | 0.004 |
| Heart failure | 13 (0.1%) | 39 (0.1%) | 0.026 | 13 (0.2%) | 37 (0.1%) | 0.029 |
| Autoimmunity disease | 15 (0.2%) | 65 (0.1%) | 0.018 | 11 (0.1%) | 55 (0.1%) | 0.012 |
| Surgery | 664 (7.6%) | 2902 (4.7%) | 0.121 | 630 (7.9%) | 2699 (4.8%) | 0.129 |
| Chemotherapy | 6660 (75.8%) | 32,301 (51.8%) | 0.515 | 5992 (75.4%) | 29,493 (52.2%) | 0.496 |
| Targeted therapy | 1516 (17.2%) | 5290 (8.5%) | 0.264 | 1437 (18.1%) | 4901 (8.7%) | 0.279 |
| Glucocorticoid | 6193 (70.5%) | 32,918 (52.8%) | 0.370 | 5689 (71.6%) | 30,102 (53.3%) | 0.384 |
| Days from baseline to ICI initiation | |  |  |  |  |  |
| Median | 40 (8, 209) | —— |  | 43 (8, 220) | —— |  |
| Mean (SD) | 168 (280) | —— |  | 174 (283) | —— |  |

^a^ up to 10 controls were matched for each exposed individual using an “exposure density sampling” approach.

^b^ SMD, standardized mean difference (shown as an absolute value). An SMD >0.1 indicated a between-group imbalance of baseline characteristics.

Abbreviation: ICI, Immune-Checkpoint-Inhibitor; SD, standard deviation; COPD, chronic obstructive pulmonary disease; PD1, programmed cell death receptor-1.

**Supplementary Table 7** Characteristics at the index date of patients taking ICI therapy or targeted therapy in sensitivity analysis 4.

|  | ICI therapy  N (%) | Targeted therapy  N (%) | SMD ^a^ |
| --- | --- | --- | --- |
| Total | 11,199 | 9297 |  |
| Gender (male) | 8364 (74.7%) | 5151 (55.4%) | 0.413 |
| Age at index date |  |  |  |
| Mean (SD) | 61 (9.87) | 59 (10.57) |  |
| Median (quartile) | 62 (55, 68) | 59 (52, 66) |  |
| Birth year |  |  |  |
| <1950 | 1577 (14.1%) | 1306 (14.0%) | 0.135 |
| 1950-1960 | 4489 (40.1%) | 3267 (35.1%) |  |
| 1960-1970 | 3755 (33.5%) | 3204 (34.5%) |  |
| >1970 | 1378 (12.3%) | 1520 (16.3%) |  |
| Family history of cancer |  |  |  |
| Yes | 808 (7.2%) | 577 (6.2%) | 0.160 |
| Missing | 747 (6.7%) | 1036 (11.1%) |  |
| Bone metastasis | 4405 (39.3%) | 3466 (37.3%) | 0.042 |
| Brain metastasis | 2463 (22.0%) | 2461 (26.5%) | 0.105 |
| Pericardial metastasis | 95 (0.8%) | 74 (0.8%) | 0.006 |
| Liver metastasis | 1864 (16.6%) | 1219 (13.1%) | 0.099 |
| Contralateral Lung metastasis | 4224 (37.7%) | 2970 (31.9%) | 0.121 |
| Adrenal metastasis | 1169 (10.4%) | 619 (6.7%) | 0.136 |
| High blood pressure | 2137 (19.1%) | 1462 (15.7%) | 0.089 |
| Diabetes | 1083 (9.7%) | 621 (6.7%) | 0.109 |
| COPD | 310 (2.8%) | 149 (1.6%) | 0.080 |
| Coronary heart disease | 109 (1.0%) | 69 (0.7%) | 0.025 |
| Myocardial infarction | 52 (0.5%) | 15 (0.2%) | 0.054 |
| Heart failure | 88 (0.8%) | 80 (0.9%) | 0.008 |
| Autoimmunity disease | 86 (0.8%) | 103 (1.1%) | 0.035 |
| Surgery | 1170 (10.4%) | 878 (9.4%) | 0.034 |
| Chemotherapy | 8538 (76.3%) | 5786 (62.2%) | 0.307 |
| Glucocorticoid | 7957 (71.1%) | 6611 (71.1%) | 0.001 |

^a^ SMD, standardized mean difference (shown as an absolute value). An SMD >0.1 indicated a between-group imbalance of baseline characteristics.

Abbreviation: ICI, Immune-Checkpoint-Inhibitor; SD, standard deviation; COPD, chronic obstructive pulmonary disease.
